# Computerized Segmentation Method for Nonmasses on Breast DCE-MRI Images Using ResUNet++ with Slice Sequence Learning and Cross-Phase Convolution

**DOI:** 10.1007/s10278-024-01053-6

**Published:** 2024-03-05

**Authors:** Akiyoshi Hizukuri, Ryohei Nakayama, Mariko Goto, Koji Sakai

**Affiliations:** 1 Department of Electronic and Computer Engineering, Ritsumeikan University, 1-1-1 Noji-Higashi, Kusatsu, Shiga 525-8577 Japan; 2 Department of Radiology, Graduate School of Medical Science, Kyoto Prefectural University of Medicine, 465 Kajiicho, Kawaramachi Hirokoji, Kamigyoku, Kyoto 602-8566 Japan

**Keywords:** Nonmass, Slice sequence learning, Cross-phase convolution, Convolutional neural network, Breast magnetic resonance imaging

## Abstract

The purpose of this study was to develop a computerized segmentation method for nonmasses using ResUNet++ with a slice sequence learning and cross-phase convolution to analyze temporal information in breast dynamic contrast material-enhanced magnetic resonance imaging (DCE-MRI) images. The dataset consisted of a series of DCE-MRI examinations from 54 patients, each containing three-phase images, which included one image that was acquired before contrast injection and two images that were acquired after contrast injection. In the proposed method, the region of interest (ROI) slice images are first extracted from each phase image. The slice images at the same position in each ROI are stacked to generate a three-dimensional (3D) tensor. A cross-phase convolution generates feature maps with the 3D tensor to incorporate the temporal information. Subsequently, the feature maps are used as the input layers for ResUNet++. New feature maps are extracted from the input data using the ResUNet++ encoders, following which the nonmass regions are segmented by a decoder. A convolutional long short-term memory layer is introduced into the decoder to analyze a sequence of slice images. When using the proposed method, the average detection accuracy of nonmasses, number of false positives, Jaccard coefficient, Dice similarity coefficient, positive predictive value, and sensitivity were 90.5%, 1.91, 0.563, 0.712, 0.714, and 0.727, respectively, larger than those obtained using 3D U-Net, V-Net, and nnFormer. The proposed method achieves high detection and shape accuracies and will be useful in differential diagnoses of nonmasses.

## Introduction

Breast cancer is the most commonly diagnosed cancer among women. In 2020, approximately 684,000 women died of breast cancer globally [1]. The early detection and treatment of breast cancer are critical. For example, Reynolds et al. [2] reported that 95% of patients were completely cured when breast cancer was detected and treated early. A nonmass where a mass has not yet formed is an important indicator for breast cancers in breast dynamic contrast material-enhanced magnetic resonance imaging (DCE-MRI) images. However, distinguishing whether a nonmass lesion is malignant or benign is difficult for radiologists [3]. For example, Baltzer et al. [4] reported that the positive predictive value (PPV) of nonmasses in DCE-MRI was quite low. Unnecessary biopsies can also cause physical problems and financial burdens on patients.

Researchers have developed computer-aided diagnosis (CADx) schemes to distinguish between benign and malignant breast nonmasses to improve the PPV of nonmasses in breast DCE-MRI. Newell et al. [5] developed a CADx scheme based on an artificial neural network with morphological, textural, and kinetic features to distinguish between benign and malignant nonmasses on DCE-MRI. Tan et al. [6] and Ayatollahi et al. [7] also used machine learning techniques with texture features on DCE-MRI images to distinguish between benign and malignant nonmasses. Li et al. [8] and Zhou et al. [9] developed computerized classification methods for benign and malignant nonmasses using radiomic features. In these methods, all features are extracted from segmented nonmass regions. Thus, it is necessary to segment the nonmasses on DCE-MRI images to evaluate the likelihood of malignancy of the nonmasses.

Cancer patterns tend to show rapid early enhancement (wash-in), followed by a loss of enhancement (wash-out) on DCE-MRI images over time [10]. Nonmasses exist in the slice images and through-plane direction in DCE-MRI images. Therefore, to segment nonmass regions accurately, it is necessary to use a computerized method to analyze the dynamic changes in the signal intensity and the relationship between consecutive slices in both lesions. The main objective of this study was to develop a computerized segmentation method for nonmasses in breast DCE-MRI images using ResUNet++ and a combination of slice sequence learning to analyze the sequential information of consecutive slices and cross-phase convolution to incorporate the dynamic changes in the lesion signal intensity. The main contributions of this study can be summarized as follows:

1. We propose a computerized segmentation method for nonmasses using ResUNet++ [11, 12] using cross-phase convolution to analyze the temporal information among DCE-MRI images acquired at different times and slice sequence learning to examine the sequential information between continuous slices.
2. We show that cross-phase convolution can analyze the temporal information, whereas slice sequence learning can analyze the sequential information between continuous slices.
3. We demonstrate that the segmentation accuracies are improved using the proposed network, ResUNet++ with the cross-phase convolution and slice sequence learning, compared with those obtained by the original ResUNet++, ResUNet++ with cross-phase convolution, ResUNet++ with slice sequence learning, 3D U-Net [13], V-Net [14], and nnFormer [15].

The remainder of this paper is organized as follows. The “Related Work” section presents an overview of related studies on the segmentation task of masses/nonmasses on DCE-MRI images. Our dataset is outlined in the “Materials” section, and a detailed explanation of the proposed method is presented in the “Methods” section. The results are described in detail in the “Experimental Results” section. The “Discussion” section provides a comparative analysis with previous methods. Finally, the conclusion and limitations of this paper are described in the “Conclusions” section.

## Related Work

As mass lesions in DCE-MRI generally have clear boundaries, they can be detected and segmented in a relatively straightforward manner. However, the specificity of masses in DCE-MRI is low, typically ranging from 30 to 70% [16, 17]. In contrast, nonmass lesions exhibit a heterogeneous appearance in DCE-MRI because the tumorous tissues and stroma are mixed. In addition, the boundaries of nonmasses are generally indistinct. Therefore, the detection and segmentation of nonmasses is extremely challenging [18]. The PPV of nonmasses has also been reported to be lower than that of masses [4, 19].

Several researchers have attempted to develop CADx schemes to distinguish between benign and malignant breast lesions [5–9]. As the first step, segmentation methods for breast lesions in DCE-MRI images have been established [9, 20–29]. These methods are primarily divided into image-processing-based and deep-learning-based methods, including convolutional neural networks (CNNs). 

In terms of image-processing-based methods, Zhou et al. [9] used a fuzzy C-means clustering method to segment mass regions. Shokouhi et al. [20] also proposed a segmentation method for masses in DCE-MRI using region growing based on the fuzzy C-means clustering method, which enables each pixel to belong to multiple classes with varying degrees of membership. However, in the aforementioned studies, regions of interest (ROIs) containing masses were required in advance. Zheng et al. [21] proposed a graph cut–based method for mass segmentation. This method segments mass regions by minimizing the energy function related to the similarity and segmentation smoothness of the pixels. One limitation of this method is that it requires manual initialization to provide the seeds or ROI for the foreground and background. 

In terms of deep learning–based methods, Carvalho et al. used SegNet [22] and U-Net [23] for mass segmentation [24]. Dalmış et al. [25] proposed a computerized segmentation method for breast and fibroglandular tissue in DCE-MRI using two consecutive U-Nets. This method first segments the breast in the entire DCE-MRI image, which is followed by segmentation of the fibroglandular tissue inside the segmented breast. Haq et al. [26] employed conditional generative adversarial networks for mass segmentation. However, one critical limitation of these studies is that two-dimensional (2D) axial slice images were only used as inputs for the networks in the DCE-MRI. Thus, these methods cannot analyze the sequential information between continuous slices. On the other hand, Khaled et al. [13] developed an automated mass segmentation method using a 3D U-Net to analyze the axial direction information. The 3D U-Net was extended by replacing 2D operations with 3D equivalents. In this method, the DCE-MRI volumes are divided into small patches of certain sizes for the 3D U-Net. Other researchers have also proposed 3D CNN-based segmentation methods [14, 27, 28]. Recently, some researchers have developed transformer-based networks for the segmentation task. The transformer can capture the global interactions between contexts. Qin et al. [29] proposed a two-stage breast mass segmentation model. In this method, the rough outline of the breast region is first segmented by U-Net. Based on the segmented rough outline of the breast region, a TR-IMUnet model is employed for accurate segmentation of the shape of masses. This model is based on U-Net. A transformer module, an improved dynamic rectified linear unit module, and a multi-scale parallel convolution fusion module are newly employed. Moreover, Zhou et al. [15] developed nnFormer using a 3D transformer for volumetric medical image segmentation. In this model, the local and global volume–based self-attention mechanism is newly introduced to the nnFormer for learning volume representation. The authors showed that the segmentation performance of the nnFormer was improved compared to those of previous segmentation models. However, these methods have numerous trainable parameters compared to 2D CNN, which is disadvantageous for smaller datasets. Most of the aforementioned studies focused on the segmentation of masses. To the best of our knowledge, few studies exist on segmentation methods for nonmasses [9]. In [9], radiologists determined the location and slice range of the nonmasses in breast DCE-MRI images. It would be tedious for clinicians to determine them manually in clinical practice.

## Materials

DCE-MRI images from February 2010 to July 2022 were acquired using a 3-T MRI scanner (Magnetom Skyra; Siemens Healthcare) with a dedicated 16-channel breast coil at the University Hospital, Kyoto Prefectural University of Medicine (Kyoto, Japan). The inclusion criteria for this study were as follows: patients who were diagnosed as having non-mass enhancement by a board-certified radiologist with 15 years of experience in breast MRI and histopathologically confirmed to be malignant via biopsy or benign via biopsy or follow-up (at least 2 years). A total of 59 consecutive patients were identified. The exclusion criteria were patients receiving any prior treatment for breast cancer (*n* = 2), insufficient image quality (*n* = 2), and breast lymphoma (*n* = 1). Finally, the database consisted of 54 DCE-MRI examinations, which contained three sequential phase images from 54 patients (mean age: 55.2 years, age range: 21–85 years).

**Fig. 1:**
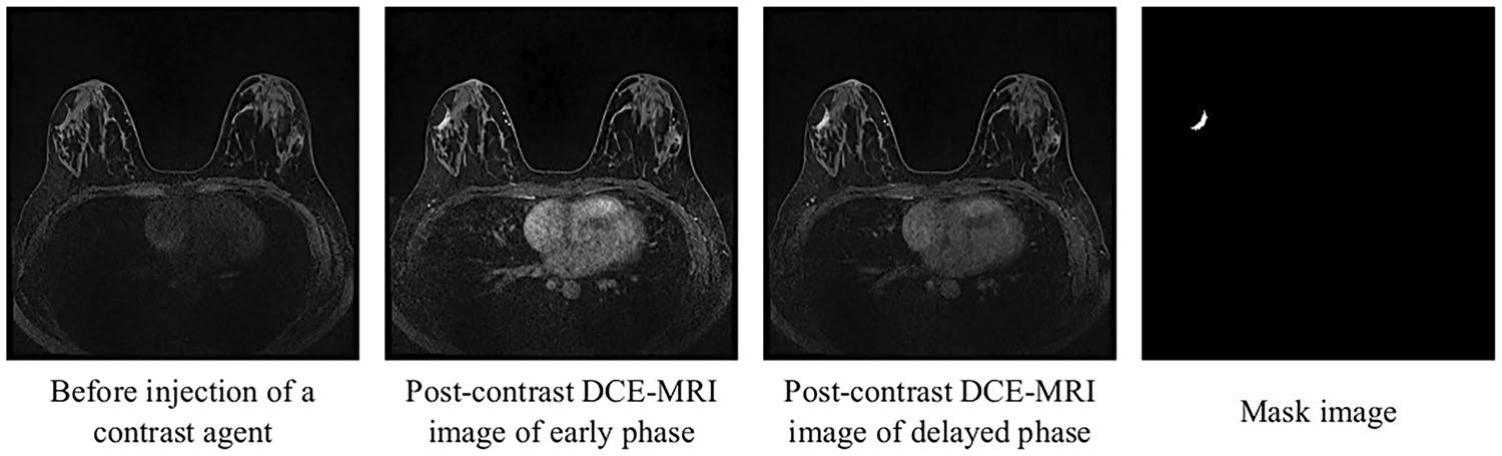
Example of pre-contrast DCE-MRI image, post-contrast DCE-MRI images, and mask image

A 3D MRI was obtained as a DCE-MRI before and two times after bolus injection of a contrast agent. Two post-contrast scans were performed with the k-space centered at 90 s (early phase) and 300 s (delayed phase) following contrast injection. The one pre-contrast and two post-contrast series generated images with a spatial resolution of 0.91 × 0.91 × 1.0 mm^3^ and a data matrix of 352 × 352 pixels. Each of the three image scan series consisted of 144 slices. A total of 63 nonmasses were included. A board-certified radiologist with 15 years of experience in breast MRI manually determined the nonmass mask images using the 3D Slicer software (https://www.slicer.org). Figure 1 shows examples of a pre-contrast DCE-MRI image, post-contrast DCE-MRI images of the early and delayed phases, and mask image.

A *k*-fold cross-validation method [30] with *k* = 5 and a patient-level split was used to train and test the proposed method. The dataset of 54 patients was randomly divided into five groups on the patient level, and the number of patients in each group was approximately equal. One group was used for the test dataset, whereas the remaining four were used for the training dataset. This process was repeated five times until each group was formed as a test dataset.

## Methods

**Fig. 2:**
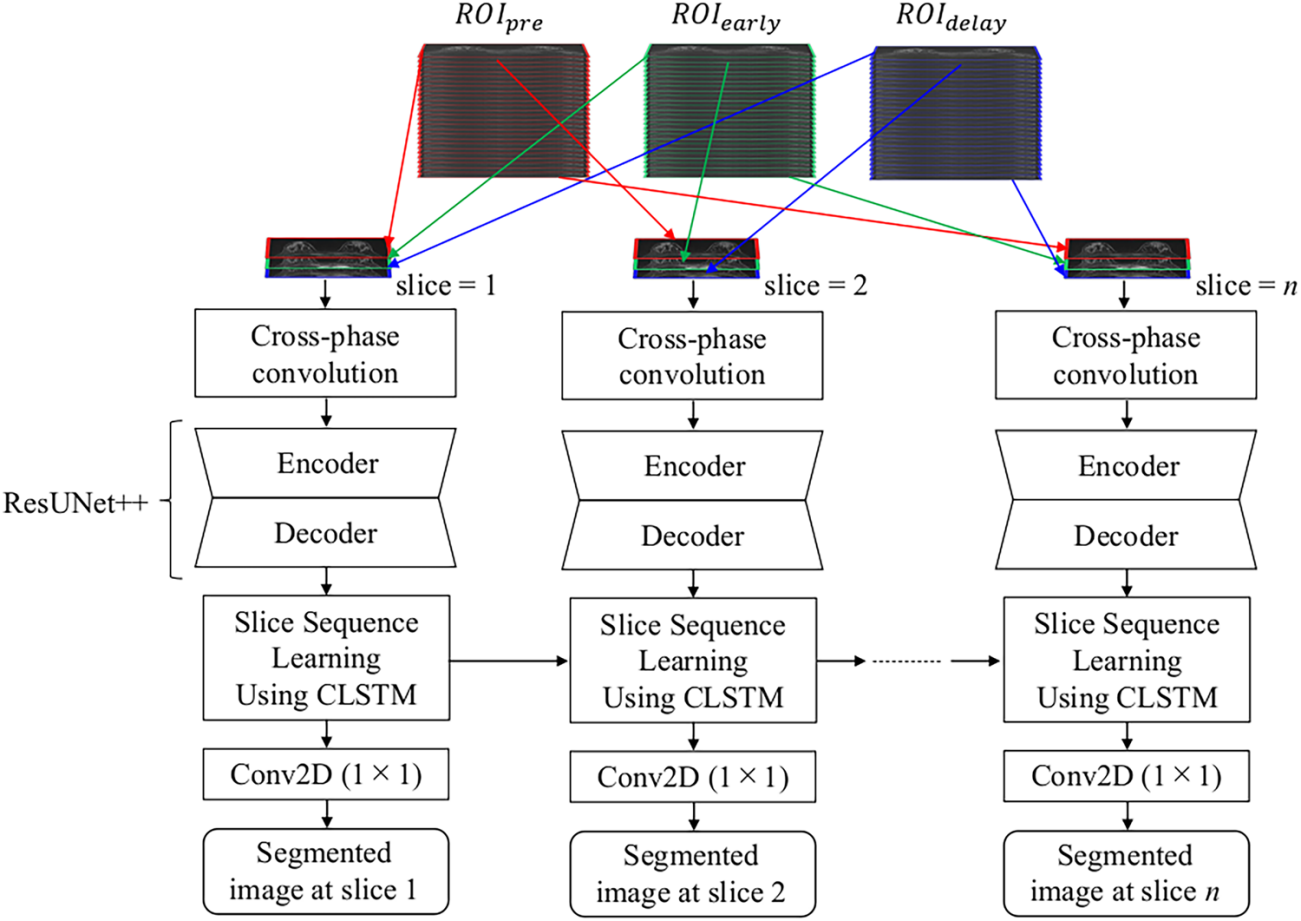
Overview of the proposed network

Figure 2 presents an overview of the proposed network, which primarily consists of three parts: ResUNet++ [11, 12], a cross-phase convolution to analyze dynamic changes in the lesion signal intensity, and slice sequence learning to analyze the sequential information for consecutive slice images in DCE-MRI. The details of the proposed network are presented in the following section.

### Data Augmentation

A CNN requires sufficient training data to achieve high segmentation accuracy. However, the number of training data in the database was limited. As a small amount of training data may cause the CNN to overfit; in this study, the amount of training data was doubled using horizontal flipping [31]. This data augmentation approach was employed only for the training datasets.

### Extraction of Breast Region

**Fig. 3:**
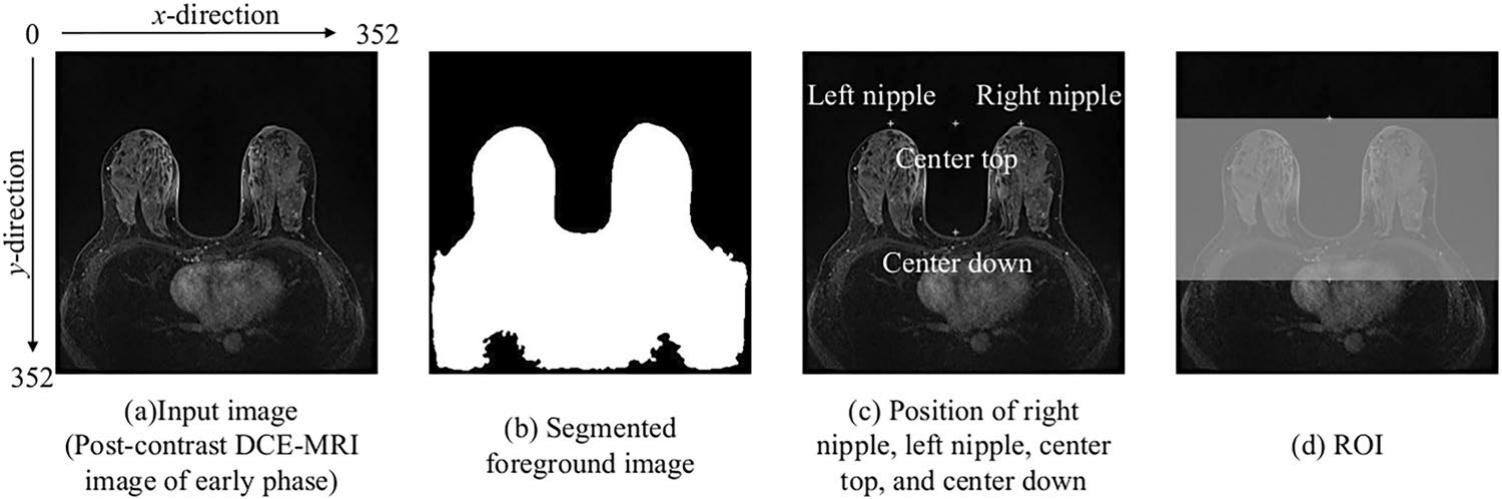
Example of foreground segmentation

The breast regions were extracted from the pre-contrast and two post-contrast DCE-MRI images to reduce the effects on other structures, such as the chest. The foreground, including the breast region, was segmented by applying a gray-level thresholding technique [31] to the post-contrast DCE-MRI early-phase images. The threshold was empirically set to a 10-pixel value. Figures 3a and b depict examples of the input and segmented foreground images, respectively. Subsequently, the position ($py$) in the $y$-direction for the nipple regions was determined using the smaller value of the left nipple position in the $y$-direction (${py}_{left}$) and the right nipple position in the $y$-direction (${py}_{right}$). The search range was 0–$W/2$ in the $x$-direction, 0–$H/2$ in the $y$-direction, and 0 for the number of slices ($S$) in the $z$-direction (through-plane), where $W$, $H$, and $S$ represent 352, 352, and 144 slice images, respectively. Conversely, the search range for the $x$-direction was set to $W$/2–$W$ to calculate the right nipple position (${px}_{right}$, ${py}_{right}$). The search ranges for the $y$- and $z$-directions were identical to those used to calculate the left nipple position. Subsequently, the top-center position (${cx}_{top}$, ${cy}_{top}$) between the left and right nipples was calculated using the following equation:

1 $$\left({cx}_{top},{cy}_{top}\right)=(\frac{{nlx}_{top}+{nrx}_{top}}{2},py)$$

The bottom center position (${cx}_{bottom}$, ${cy}_{bottom}$) of the breastbone with the minimum value in the $y$-direction was determined based on the top center position according to the segmented breast region using the raster scan technique. The search range was ${cx}_{top}$ in the $x$-direction, ${cy}_{top}$–$H$ in the $y$-direction, and 0–$S$ in the z-direction. Figure 3c shows an example of the left nipple, right nipple, top center, and bottom center positions. The cropping area position for the breast region was determined in the range of 1–$W$ in the $x$-direction, (${cy}_{top}$-${interval}_{top}$)–(${cy}_{bottom}$+${interval}_{down}$) in the $y$-direction, and 1–$S$ in the $z$-direction. In this case, ${interval}_{top}$ and ${interval}_{down}$ were empirically set to 5 and 50 pixels, respectively. Based on the position, ${ROI}_{pre}$, ${ROI}_{early}$, and ${ROI}_{post}$ including the breast region were extracted from the pre- and post-contrast DCE-MRI images of the early and delayed phases, respectively. Figure 3d shows the clipping area range in light white. Each ROI was resized to 192 × 352 pixels.

### Cross-Phase Convolution

The cross-phase convolution, which consisted of a 3D convolutional layer, rectified linear unit (ReLU) function, and batch normalization layer, was developed to obtain the optimal fusion among ROIs that were acquired at different times through the network training. Slice images of the same position from the ROIs at the pre-, early, and delayed phases were first stacked together to generate a 3D tensor. The size of the 3D tensor was three phases × 192 × 352 pixels. The 3D convolution layer, ReLU function, and batch normalization layer were sequentially applied to the 3D tensor. The 3D convolution layer kernel size was 3 × 1 × 1, where 3 is the number of phases. The number of filters in the 3D convolution layer was 16. This 3D layer was designed to assign weights to each ROI by training and summing their values. New feature maps were generated based on optimal fusion among the ROIs acquired at different times by cross-phase convolution.

### ResUNet++ 

Our database, which included 54 patient examinations, was relatively small. Jha et al. [12] showed that ResUNet++ worked well with a smaller number of images. In their experiments, ResUNet++ also outperformed the well-known segmentation architectures U-Net and ResUNet. Therefore, ResUNet++ was used as the baseline network to segment nonmasses in DCE-MRI images.

**Fig. 4:**
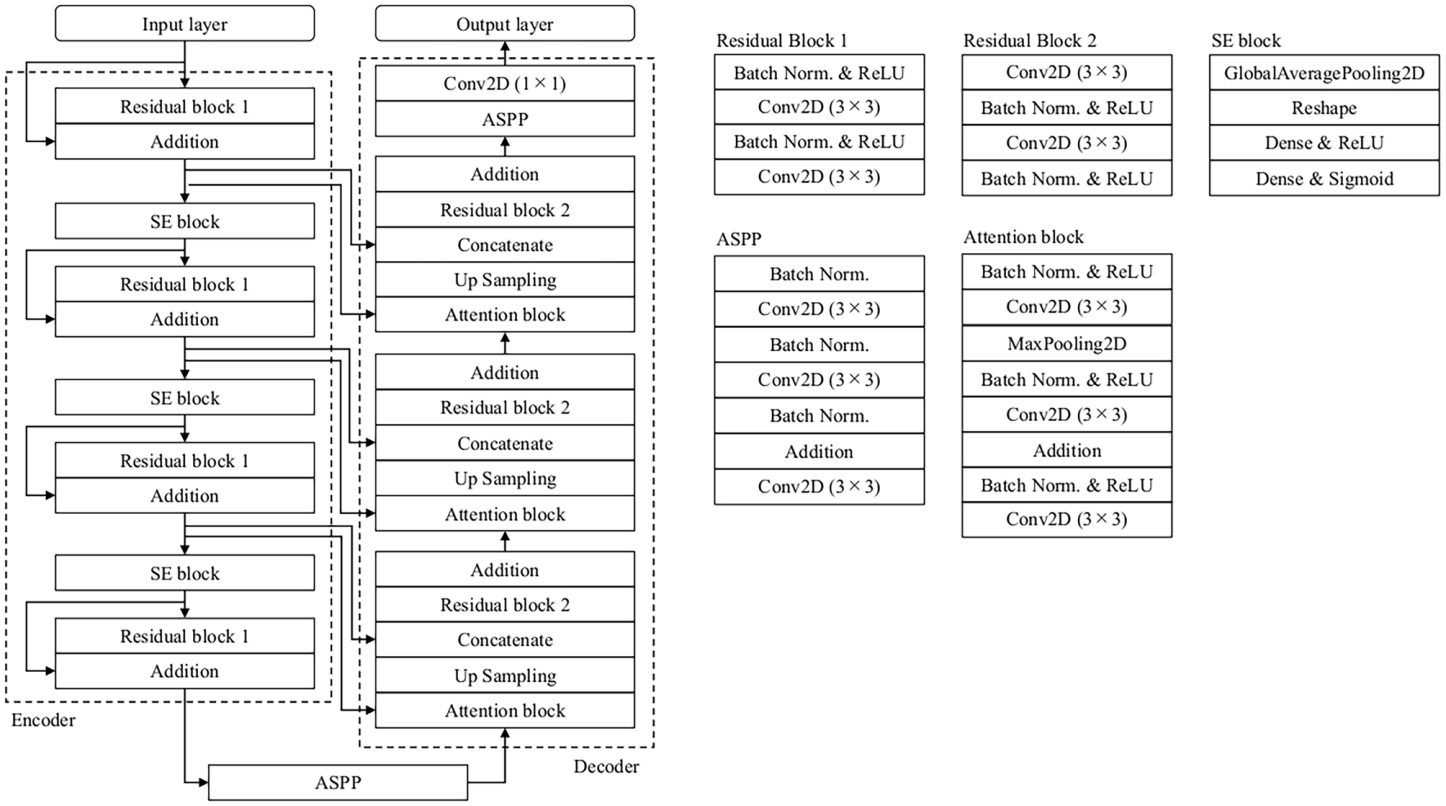
Architecture of ResUNet++

ResUNet++ contained four important components: a residual block, a squeeze and excitation (SE) block, atrous spatial pyramid pooling (ASPP), and an attention block. Figure 4 shows the ResUNet++ architecture employed in this study. This network had an input layer, four encoder blocks, an SE block, ASPP, three decoder blocks, and an output layer. The feature maps that were generated by the cross-phase convolution were first input into ResUNet++. Each residual block consisted of two batch normalization layers, a ReLU function, and convolutional layers. Note that only the first residual block consisted of two convolutional layers: a batch normalization layer and a ReLU function. Skip connections connected the input and output of the residual blocks to prevent the vanishing gradient problem. The residual block outputs, excluding the final residual block, were fed into the SE blocks. The SE block learned the importance of different feature channels and adaptively recalibrated them. The feature maps obtained by the encoders were passed through the ASPP, which acted as a bridge between the encoder and decoder and could effectively aggregate contextual information at different scales without increasing the computational cost. The ASPP output was input into the decoder. The decoder consisted of attention and residual blocks and the ASPP. The attention block, which determined which parts of the images the network focused on, was executed before each residual block. The generated feature maps that were obtained by each attention block were upsampled by the nearest neighbor and concatenated with feature maps from their corresponding encoding path. The decoder output was fed to the ASPP. Subsequently, the nonmasses were segmented by applying a 1 × 1 convolutional layer to the 64-component feature vector that was obtained by the final ASPP.

### Slice Sequence Learning

Convolutional long short-term memory (CLSTM) [32, 33] was introduced after the decoder in ResUNet++ to analyze the sequential information between consecutive slices. Compared to traditional LSTM, CLSTM replaces matrix multiplication with a convolutional operator to preserve long-term spatial information. The feature maps that were obtained from the second convolutional layer in the decoder were used as inputs for the CLSTM. The CLSTM consisted of an input gate ${i}_{t}$, forget gate ${f}_{t}$, memory cell ${C}_{t}$, output gate ${o}_{t}$, and hidden state ${h}_{t}$, as follows:

1 $${i}_{t}=\upsigma \left({W}_{xi}*{x}_{t}+{W}_{hi}*{h}_{t-1}+{b}_{i}\right)$$

2 $${f}_{t}=\upsigma \left({W}_{xf}*{x}_{t}+{W}_{hf}*{h}_{t-1}+{b}_{f}\right)$$

3 $${C}_{t}={f}_{t}\circ {C}_{t-1}+{i}_{t}{\text{tanh}}\left({W}_{xc}*{x}_{t}+{W}_{hc}*{h}_{t-1}+{b}_{c}\right)$$

4 $${o}_{t}=\upsigma \left({W}_{xo}*{x}_{t}+{W}_{ho}*{h}_{t-1}+{b}_{o}\right)$$

5 $${h}_{t}={o}_{t}\circ {\text{tanh}}\left({C}_{t}\right)$$

where ${x}_{t}$, ${h}_{t}$, and ${C}_{t}$ are the input, hidden state, and memory cell tensors, respectively, at time step $t$. In this study, the time steps represented the DCE-MRI slice images. ${b}_{i}$, ${b}_{f}$, ${b}_{o}$, and ${b}_{c}$ are biased terms. ${W}_{x*}$ and ${W}_{h*}$ are the convolutional kernels for the input and hidden states, respectively. The CLSTM with feature maps obtained by the second-last convolutional layer in the decoder was employed to learn the consecutive slice sequential information. Subsequently, a convolutional layer with a kernel of size 1 × 1 was employed to map the feature maps that were obtained from the CLSTM to a binary output image (nonmass region: 1, other: 0).

### Loss Function for Training Proposed Network

The proposed network loss function is shown in Eq. (6):

6 $$\mathcal{L}={TL}_{ResUNet++}+ {TL}_{CLSTM}$$

where ${TL}_{ResUNet++}$ is defined as the Tversky loss [34] between the output images by ResUNet++ and the mask images and ${TL}_{CLSTM}$ is defined as the Tversky loss between the image output by the CLSTM and the mask images. The Tversky loss function [34] is expressed as

7 $$TL=1- \frac{\sum_{i=1}^{N}{p}_{i}{g}_{i}}{{\sum }_{i=1}^{N}{p}_{i}{g}_{i}+\alpha {\sum }_{i=1}^{N}\left(1-{g}_{i}\right){p}_{i}+\beta {\sum }_{i=1}^{N}{g}_{i}\left(1-{p}_{i}\right)}$$

where ${p}_{i}$ and ${g}_{i}$ denote the nonmass regions segmented by the proposed network and the mask images at pixel $i$, respectively; $N$ is the number of image pixels; and $\alpha$ and $\beta$ are hyperparameters that control the tradeoff between false positives and negatives.

### Comparison with Other Segmentation Networks

We compared the proposed network with a network using a space–time memory (STM) [35] and the cross-phase convolution (${{\text{network}}}_{STM\_CPC}$). The STM calculates the spatio-temporal attention on every pixel in multiple slice images of DCE-MRI [35]. Here, the baseline network of the ${{\text{network}}}_{STM\_CPC}$ was the same as the proposed network.

The proposed network was also compared with 3D-based CNN models, namely 3D U-Net,V-Net, and nnFormer. In 3D U-Net and V-Net, ${ROI}_{pre}$, ${ROI}_{early}$, and ${ROI}_{delay}$ were first divided into 64 × 64 × 64 patches (small regions), and the mask images were divided identically at the corresponding positions. Patches in ${ROI}_{pre}$, ${ROI}_{early}$, and ${ROI}_{delay}$ were used as the input layer in each network for training. The patches obtained from the mask images were used as the desired output values in the network output layer. Here, in nnFormer, ${ROI}_{pre}$, ${ROI}_{early}$, and ${ROI}_{delay}$ were divided into 96 × 96 × 96 patches (small regions), and the mask images were divided identically at the corresponding positions.

### Proposed Network Training and Testing

The proposed network was developed and evaluated using PyTorch 1.10.0 on a workstation (CPU: Intel Core i9-9900X processor, RAM: 128 GB, and GPU: NVIDIA GeForce RTX 2080 Ti). Adam was employed to minimize the loss between the output values of the proposed network and mask images. In this case, ${\beta }_{1}$ and ${\beta }_{2}$ in Adam were 0.9 and 0.999, respectively. The hyperparameters for training the proposed network were set to an epoch number of 20, an initial learning rate of 1 × 10^–4^, and a mini-batch size of 5. The $\alpha$ and $\beta$ values in the loss function were set to 0.3 and 0.7, respectively. The same parameter values were used for ${{\text{network}}}_{STM\_CPC}$, 3D U-Net, V-Net, and nnFormer.

### Evaluation of Detection and Shape Accuracy

The detection and shape accuracies of the proposed network were evaluated using the ensemble average from the test datasets over the five cross-validation methods. When the gravity of a true nonmass region determined by a radiologist was within the segmented candidate for nonmasses by the proposed network, this candidate was considered to be “truly” detected. In contrast, when a true nonmass region was not within a segmented candidate, the candidate was considered to be a false positive. The Jaccard coefficient (JC), PPV, sensitivity, and Dice similarity coefficient (DSC) were used to evaluate the shape accuracy of the segmented nonmass regions using the proposed network. These evaluation criteria are defined as follows:

8 $${\text{JC}}=\frac{|A\cap B|}{|A\cup B|}$$

9 $$PPV=\frac{|A\cap B|}{|A|}$$

10 $$Sensitivity= \frac{|A\cap B|}{|B|}$$

11 $$DSC= \frac{2|A\cap B|}{\left|A\right|+|B|}=\frac{2\cdot PPV\cdot Sensitivity}{PPV+Sensitivity}$$

where *A* represents the nonmass regions segmented by the proposed network, and *B* represents the mask images. The DSC, which is also known as the F1 score, also evaluates the PPV harmonic mean and sensitivity [36].

## Experimental Results

### Ablation Study

Ablation studies were conducted to investigate the effectiveness of the cross-phase convolution and slice sequence learning in the proposed network. The experimental results are listed in Table 1. The sensitivity of ResUNet++ with cross-phase convolution (0.781) was slightly lower than that of the original ResUNet++ (0.797). However, the JC, PPV, and DSC, which indicate the harmonic mean of the PPV and sensitivity, of ResUNet++ with cross-phase convolution were improved compared to those of the original ResUNet++. The detection accuracy was identical for the original ResUNet++ and ResUNet++ with cross-phase convolution. We adopted slice sequence learning for ResUNet++ to obtain the feature representations between continuous slices. Although the sensitivity of ResUNet++ with slice sequence learning (0.757) was lower than that of the original ResUNet++ (0.797); it achieved a detection accuracy of 1.59%, DSC of 3.28%, and PPV of 7.23%. The number of false positives in ResUNet++ with cross-phase convolution (3.18) and ResUNet++ with slice sequence learning (2.22) was higher than that of the original ResUNet++ (2.17). Finally, the sensitivity of ResUNet++ with both cross-phase convolution and slice sequence learning (0.727, proposed network) was lower than that of the original ResUNet++ (0.797), that with cross-phase convolution (0.781), and that with slice sequence learning (0.757). However, the remaining evaluation indices of the proposed network improved substantially, as presented in Table 1. 

**Fig. 5:**
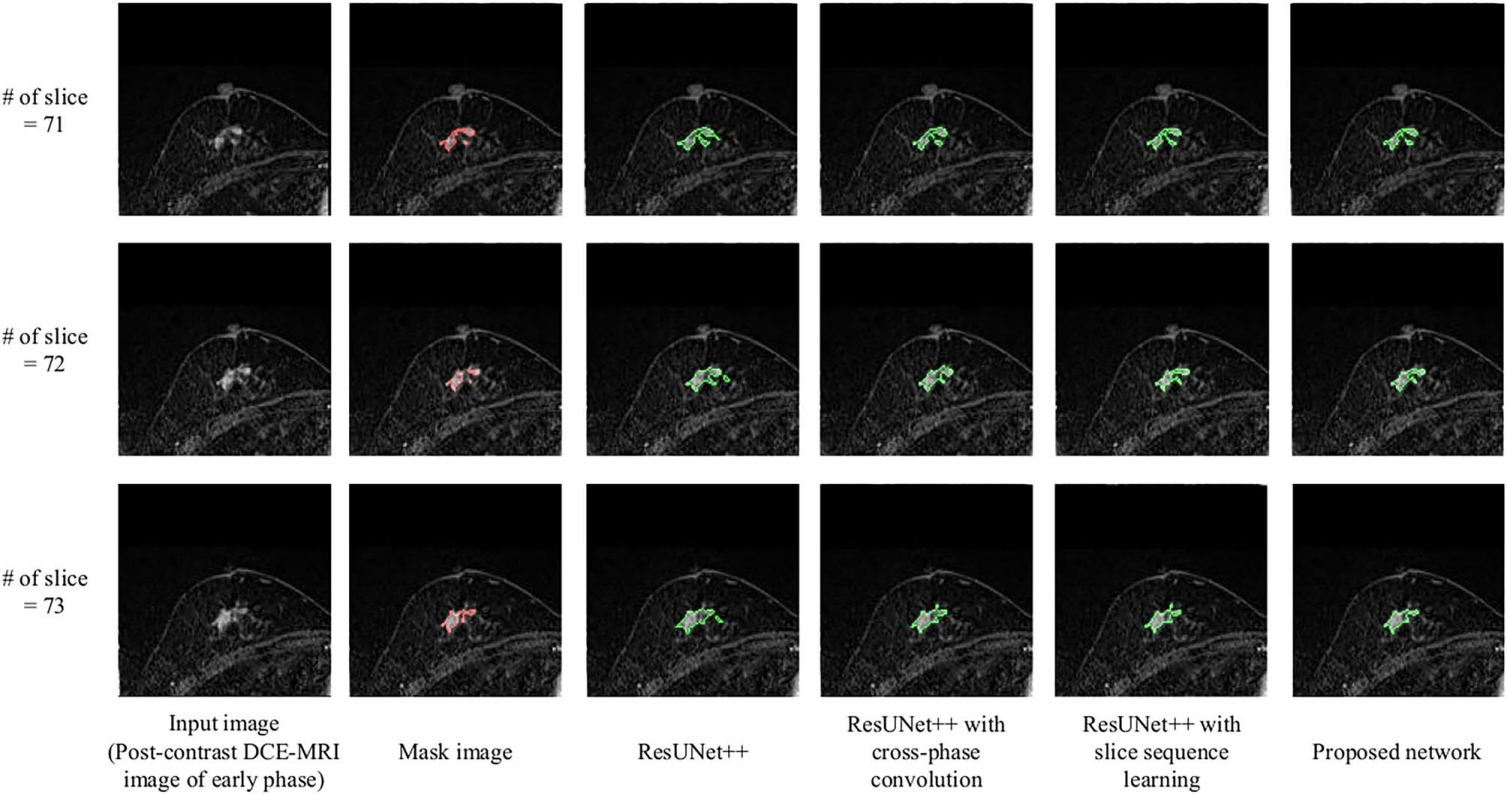
Examples of nonmasses segmented by ResUNet++, ResUNet++ with cross-phase convolution, ResUNet++ with slice sequence learning, and the proposed network

Figure 5 compares the nonmass region images segmented by the original ResUNet++, ResUNet++ with cross-phase convolution, ResUNet++ with slice sequence learning, and the proposed network. The segmented regions for the original ResUNet++, ResUNet++ with cross-phase convolution, and ResUNet++ with slice sequence learning included parts of the normal tissue that were misclassified as nonmasses. However, the proposed network correctly segmented nonmasses compared to the other networks.

**Table 1:** Ablation study results

Backbone	Cross-phase convolution	Slice sequence learning	Detection accuracy	Number of false positive per patient	JC	DSC	PPV	Sensitivity
ResUNet++	-	-	87.3% (55/63)	2.17	0.479	0.647	0.545	0.797
	✓		87.3% (55/63)	3.19	0.515	0.692	0.620	0.781
		✓	88.9% (56/63)	2.22	0.529	0.680	0.617	0.757
	✓	✓	**90.5% (57/63)**	**1.91**	**0.563**	**0.712**	**0.714**	**0.727**

### Comparison Results of Conventional Segmentation Networks

**Fig. 6:**
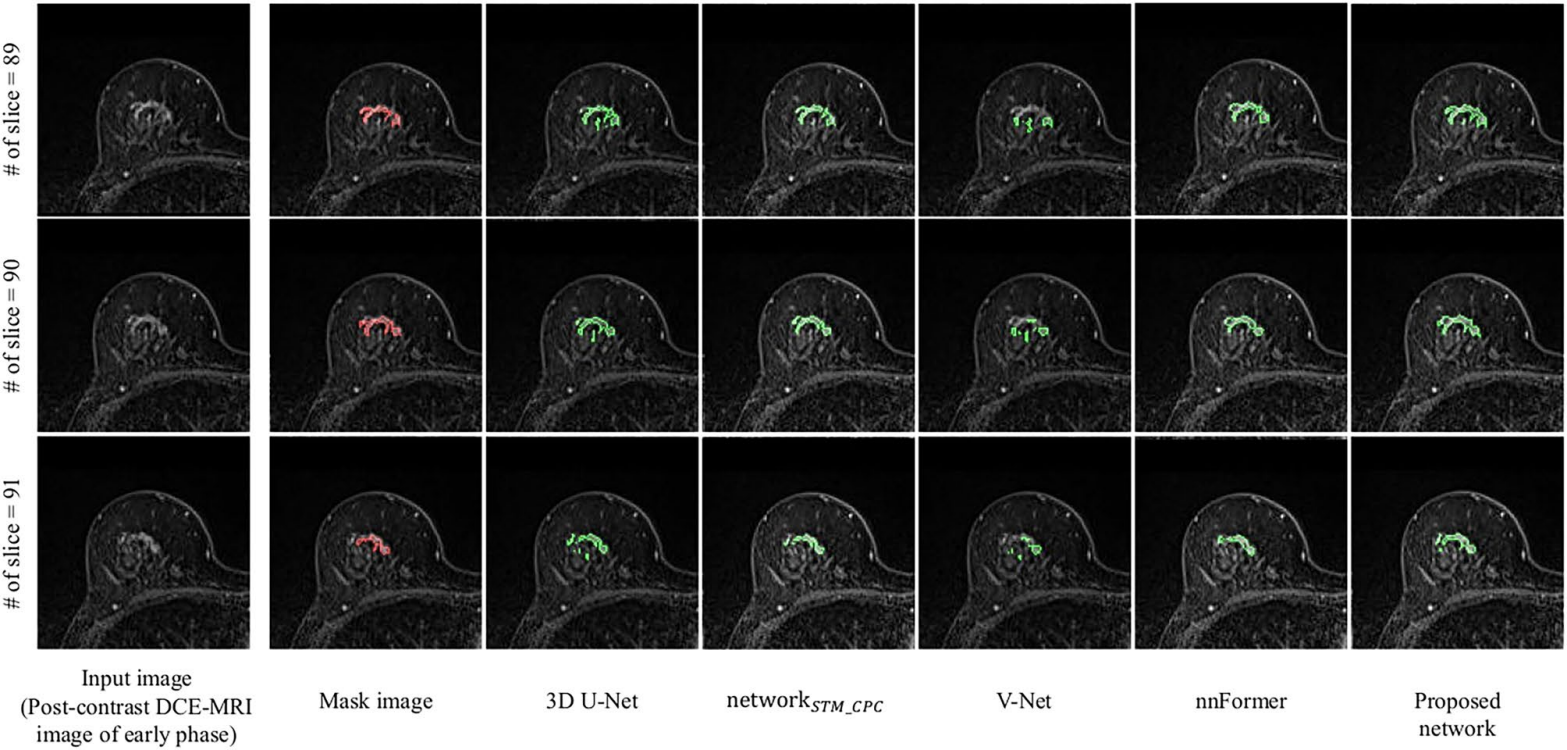
Examples of segmented nonmasses by ${{\text{network}}}_{STM\_CPC}$, 3D U-Net, V-Net, nnFormer, and the proposed network

Figure 6 shows an example of segmented nonmass regions using the proposed network, ${{\text{network}}}_{STM\_CPC}$, 3D U-Net, V-Net, and nnFormer. Table 2 shows the comparison results for ${{\text{network}}}_{STM\_CPC}$, 3D U-Net, V-Net, nnFormer, and the proposed network.

**Table 2:** Comparison of results obtained by ${{\text{network}}}_{STM\_CPC}$, 3D U-Net, V-Net, nnFormer, and the proposed network

Method	Detection accuracy	Number of false positive per patient	JC	DSC	PPV	Sensitivity
${{\text{network}}}_{STM\_CPC}$	88.9% (56/63)	4.81	0.468	0.610	0.668	0.707
3D U-Net	82.5% (52/63)	1.93	0.463	0.654	0.694	0.720
V-Net	90.5% (57/63)	3.76	0.479	0.661	0.668	0.742
nnFormer	85.7% (54/63)	2.13	0.489	0.656	0.669	0.652
**Proposed network**	**90.5% (57/63)**	**1.91**	**0.563**	**0.712**	**0.714**	**0.727**

The evaluation indices of the proposed network (90.5% detection accuracy, 1.91 false positives, 0.563 JC, 0.712 DSC, 0.714 PPV, and 0.727 sensitivity) were improved compared to ${{\text{network}}}_{STM\_CPC}$ (88.9%, 4.81, 0.468, 0.610, 0.668, and 0.707). In ${{\text{network}}}_{STM\_CPC}$, the number of training images may have been relatively small for the STM because, in [35], many of the training data compared to this study were used to train the STM. Therefore, the segmentation accuracy of ${{\text{network}}}_{STM\_CPC}$ may be improved by using more training data. However, collecting a large number of DCE-MRI images containing nonmasses is generally difficult.

In the comparison of the proposed network with 3D-based CNN models, the sensitivity of the proposed network (0.727) was lower than that of V-Net (0.742). In contrast, the remaining evaluation indices of the proposed network (90.5% detection accuracy, 1.91 false positives, 0.563 JC, 0.712 DSC, and 0.714 PPV) were higher than those of 3D U-Net (82.5%, 1.93, 0.463, 0.654, and 0.694, respectively), V-Net (90.5%, 3.76, 0.479, 0.661, and 0.668, respectively), and nnFormer (85.7%, 2.13, 0.489, 0.656, and 0.669, respectively). These 3D-based CNN models cannot enhance the regions for temporal enhancement changes in the DCE-MRI images. Therefore, we believe that the proposed network is more appropriate for segmenting non-masses on breast MRI images.

## Discussion

In this study, we developed a method to improve the segmentation performance of nonmasses in DCE-MRI images. Cross-phase convolution is used to analyze the temporal information among DCE-MRI images acquired at different times, and slice sequence learning is utilized to examine the sequential information between continuous slices. Segmented images of nonmass regions can be generated with higher accuracy than those obtained using conventional methods by employing the proposed method.

According to Table 1 and 2, the detection accuracy, JC, and DSC of the original ResUNet++ were lower than those of V-Net. Nonmasses existed in the slice images as well as in the through-plane direction in the DCE-MRI. Therefore, the original ResUNet could not capture the volumetric information of nonmass lesions.

**Fig. 7:**
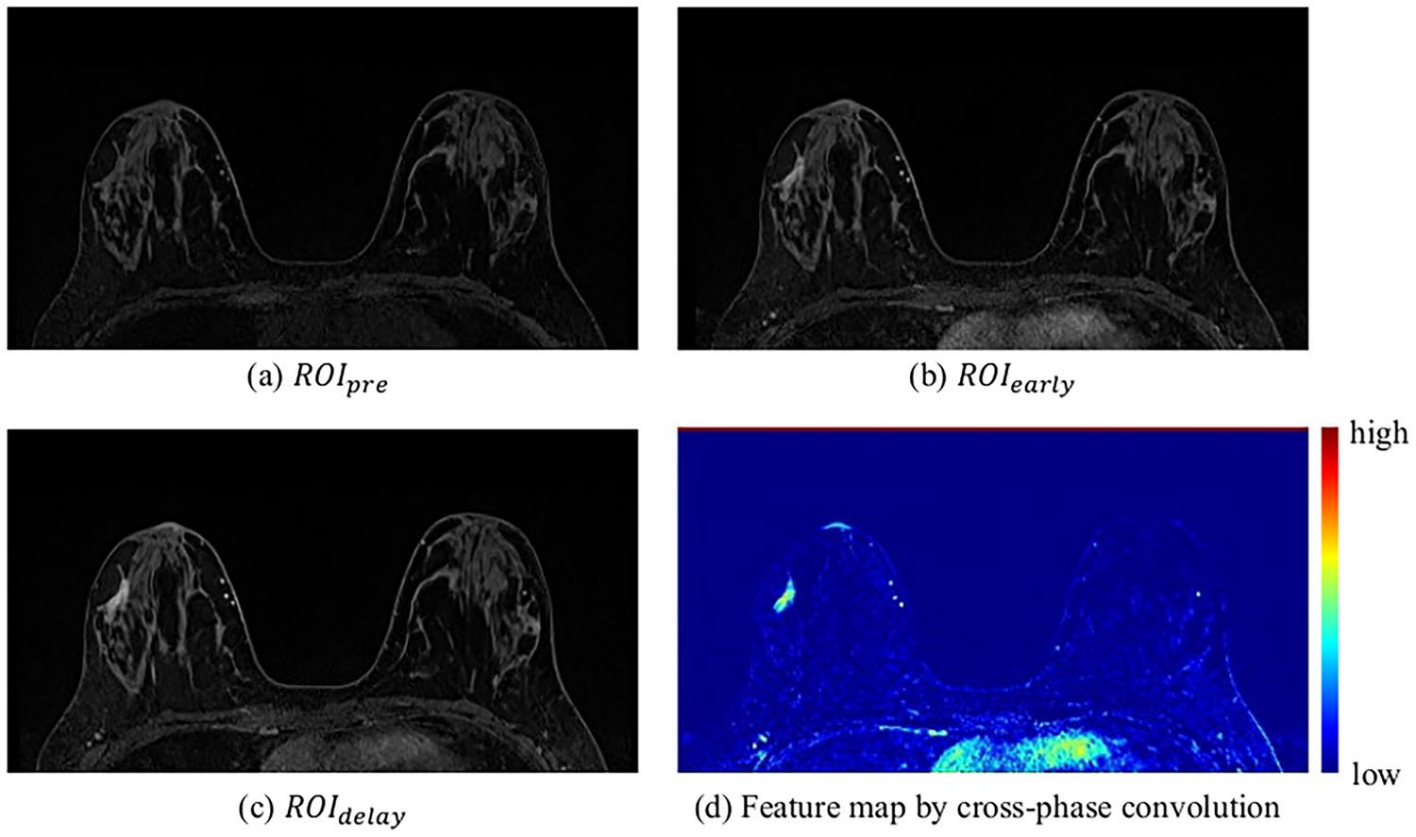
Visualization of feature map from cross-phase convolution

We compared ResUNet++ with the cross-phase convolution to the original ResUNet++ to investigate the benefits of the cross-phase convolution. As shown in Table 1, the sensitivity of ResUNet++ with cross-phase convolution was slightly lower than that of the original ResUNet++, whereas the PPV of ResUNet++ with cross-phase convolution was higher than that of the original ResUNet++. The DSC, which evaluated the balance between the sensitivity and PPV of ResUNet++ with cross-phase convolution, was higher than that of the original ResUNet++. The remaining evaluation indices for ResUNet++ with cross-phase convolution were also improved compared with those of the original ResUNet++. The network must learn the temporal enhancement changes of the nonmass regions to segment nonmass regions accurately. The original ResUNet++ could not be trained to focus on temporal enhancement changes in the nonmasses. By introducing cross-phase convolution to the original ResUNet++, the network could be trained by focusing on mass-enhancement changes that appeared in the DCE-MRI. Figure 7 shows the feature map with the highest mean value in the nonmass region among the feature maps that were obtained from the cross-phase convolution. It can be observed that the cross-phase convolution enhanced the regions for temporal enhancement changes in the DCE-MRI images. Some studies have used the difference images obtained by subtracting the pre-contrast DCE-MRI images from the post-contrast DCE-MRI images as the network input to reflect the mass enhancement changes in the network [13]. However, the different images enhanced minute differences, including noise, between the DCE-MRI images that were acquired at different times. Therefore, the use of different images may have a negative impact on the network training.

**Fig. 8:**
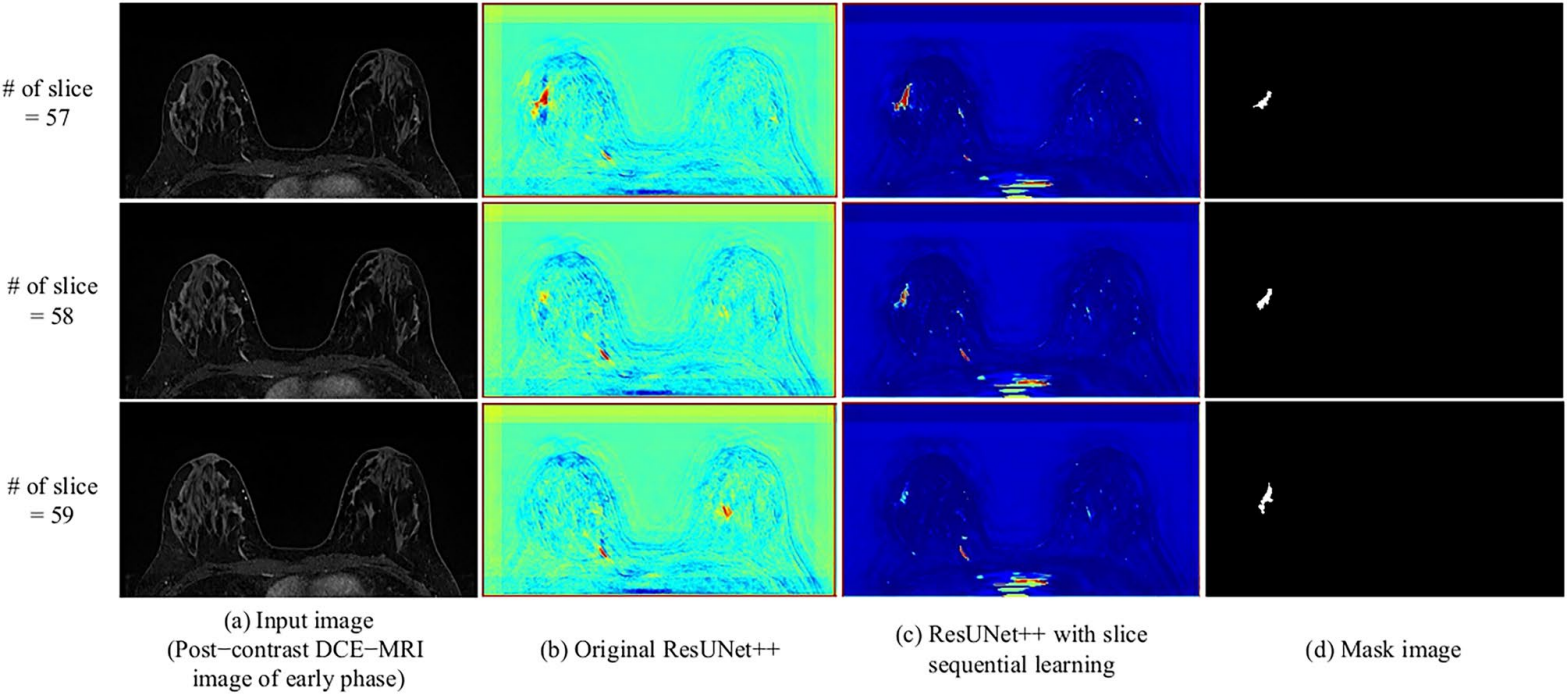
Visualization of feature maps from CLSTM and output of decoder in original ResUNet++

ResUNet++ with slice sequence learning was compared with the original ResUNet++ in terms of the detection and shape accuracy to investigate the benefits of slice sequence learning in analyzing the sequential information between consecutive slices. Although the sensitivity of ResUNet++ with slice sequence learning was lower than that of the original ResUNet++, the remaining evaluation indices for ResUNet++ with slice sequence learning were improved. Figure 8 shows the feature maps that were obtained using the CLSTM. The mean value of each feature map was calculated and visualized with the highest mean value in the nonmass region among all feature maps. The results showed that the CLSTM enhanced the features between consecutive slices that contained nonmasses compared to the original ResUNet++. Therefore, analysis of the through-plane direction enables the network to consider 3D contextual information, which assists the network in better distinguishing between nonmasses and normal tissue, especially for nonmasses that appear similar to normal tissue when viewed in a single slice.

Some limitations of this study should be noted. One is that the JC of the proposed network was relatively low. It is well known that the detection and segmentation of nonmasses are extremely challenging for radiologists because of their poorly defined boundaries compared to masses. If radiologists slightly revise the segmented nonmass images that are obtained by the proposed network, we believe that these images can be used in CADx schemes to evaluate the likelihood of malignancy of nonmasses. Therefore, when radiologists use CADx schemes, the proposed method can decrease the burden compared to manual tracing. Another limitation is that hyperparameters such as the mini-batch size, epoch number, and learning rate in the proposed network may not have been the most appropriate combination for the detection and segmentation of nonmass regions. Thus, the detection and segmentation accuracy may be improved by using more suitable hyperparameter combinations. Finally, data from only 54 patient examinations were used in this study. Therefore, future research will focus on expanding the database and evaluating the performance of the proposed network on this basis.

## Conclusions

We developed a computerized segmentation method for nonmasses in breast DCE-MRI using ResUNet++ combined with slice sequence learning and cross-phase convolution. The experiment results showed that the slice sequence learning analyzes the sequential information of consecutive slices, and the cross-phase convolution can capture the dynamic changes in the lesion signal intensity.

The proposed network exhibited a higher segmentation accuracy than the original ResUNet++, ResUNet++ with cross-phase convolution, and ResUNet++ with slice sequence learning. It also outperformed 3D U-Net, V-Net, and nnFormer. Thus, the proposed network may be useful for segmenting nonmasses in breast DCE-MRI.

Some CADx schemes for evaluating the likelihood of malignancy of nonmasses require nonmass mask images. Although radiologists manually determine the mass regions using the CADx schemes, it would be tedious for them to trace masses manually in clinical practice. Thus, when radiologists utilize CADx schemes, the proposed method can save time compared to manual tracing.

Future works will include improving the segmentation accuracy of the proposed network by optimizing the hyperparameters using Bayesian optimization. We also will introduce the proposed method into CADx schemes to evaluate the likelihood of malignancy of nonmasses and whether the classification performance is improved compared to previous segmentation methods. Moreover, we will focus on expanding the database and evaluating the performance of the proposed network on this basis.

## Data Availability

All breast DCE-MRI images in this study are owned by University Hospital Kyoto Prefectural University of Medicine, Kyoto, Japan, and cannot be made publicly available owing to patient privacy and ethical concerns.
